# Healthcare Service Quality Evaluation in a Community-Oriented Primary Care Center, Italy

**DOI:** 10.3390/healthcare11172396

**Published:** 2023-08-25

**Authors:** Andrea Ceccarelli, Alice Minotti, Marco Senni, Luca Pellegrini, Giuseppe Benati, Paola Ceccarelli, Andrea Federici, Silvia Mazzini, Chiara Reali, Francesco Sintoni, Davide Gori, Marco Montalti

**Affiliations:** 1 Operative Unit of Hygiene and Public Health of Forlì-Cesena, Department of Public Health, Romagna Local Health Authority, 47521 Cesena, Italy; 2 Nursing Unit, Primary Care and Community Medicine Department of Forlì-Cesena, Romagna Local Health Authority, 47521 Cesena, Italy; 3 Primary Care and Community Medicine Department of Forlì-Cesena, Romagna Local Health Authority, 47121 Forlì, Italy; 4 Nursing Unit, Primary Care and Community Medicine Department of Forlì-Cesena, Romagna Local Health Authority, 47121 Forlì, Italy; 5 Rubicone Health District, Romagna Local Health Authority, 47522 Cesena, Italy; 6 Hygiene Unit, Department of Biomedical and Neuromotor Sciences, University of Bologna, 40126 Bologna, Italy

**Keywords:** healthcare services, quality, perceptions, Casa della Comunità, COPD

## Abstract

Community-oriented primary care (COPC) is an inclusive healthcare approach that combines individual care with a population-based outlook, striving to offer effective and equitable services. This study concentrates on assessing the perceived quality of a “Casa della Comunità” (CdC) implemented by the Romagna Local Health Authority, which embraces the COPC model. Through the examination of user experiences, the study aims to comprehend the influence of the CdC’s care delivery model on the community’s perception of service quality. From 13–18 March 2023, paper questionnaires were distributed by trained healthcare professionals and volunteers. The cross-sectional study enrolled participants aged 18 or older, capable of understanding written Italian, and willing to take part voluntarily. A total of 741 questionnaires were collected, resulting in an overall acceptance rate of 85.6%. Among the respondents, 37.9% were female, with an average age of 55.4 ± 16.2 years. While the respondents generally held a positive view of the quality, the results displayed varying levels of satisfaction across the different areas. Multivariate analysis revealed significant associations between factors such as gender, employment status, financial resources, education level, and distance from the healthcare center with the perceived quality of the facility in terms of accessibility, environment, staff, continuity of care, and overall satisfaction. The study yielded valuable insights, identifying strengths and areas for improvement and underscoring the importance of ongoing monitoring studies to enhance patient satisfaction continuously.

## 1. Introduction

Community-oriented approaches to healthcare, which combine individual care with a population-based perspective, have been widely embraced in various countries, including the United States [1,2] and other nations [3,4]. These approaches aim to provide more effective, equitable, and efficient healthcare services by emphasizing community-oriented primary care delivery [5,6].

Community-oriented primary care (COPC) is a recognized model that focuses on delivering care to a well-defined population based on their assessed needs. The objective is to enhance the health status of the community by integrating primary care and population health [7]. COPC operates on several principles, including being accountable for comprehensive care, addressing health needs and their determinants, prioritizing needs to implement health programs, and encouraging community participation [8]. The development process of COPC involves defining and characterizing the geographic community, identifying health needs, determinants, and resources, prioritizing identified health problems, developing and implementing intervention programs, conducting surveillance and evaluation, and reassessing health needs [9]. The COPC paradigm strives to cultivate patient empowerment as a pivotal constituent within the healthcare framework concerning their personal well-being. The incorporation of cancer screening programs [10] or chronic conditions monitoring [11,12] stands to gain from this holistic COPC approach, which fundamentally reconceptualizes the patient from a passive entity within the system to an active and invested participant in the health domain. COPC achieves this transformation by dismantling historical impediments that have traditionally hindered healthcare accessibility, such as limited information dissemination, deficient health literacy, and the undervaluing of preventive medicine.

Patient experience is widely recognized as a crucial aspect of quality healthcare, alongside clinical effectiveness and patient safety. It serves as a vital indicator for assessing patient-centeredness, which refers to care that respects and responds to patient preferences, needs, and values. Evaluation of patient experience is increasingly utilized to assess the quality of care in peripheral healthcare facilities, particularly in primary care [13].

This study focuses on investigating the perceived quality within one of the Community Health Centres (CdCs) of the Romagna Local Health Authority (LHA). The survey tool used in this study is built upon previous research conducted in the region regarding perceived quality [14] and specifically targets the CdC.

The Romagna LHA serves a population of 1,114,613 individuals and currently operates 30 CdCs. This specific selection of the CdC involved in the study was based on both the fact that it was one of the first establishments in the area and its exclusion from the previous regional survey conducted a few years ago [14]. The CdC serves a catchment area encompassing approximately 60,000 users from various municipalities. Initially, the CdC was a charitable hospital dating back to the 14th century. After the establishment of the Italian National Health Service in 1978, it became a vital component of the LHA. Subsequently, it was transformed into “Health Centres” (known as ‘Case della salute’) in 2016 and eventually adopted the current model of Community Health Centres. This transition introduced a new organizational model for delivering healthcare services, employing numerous professionals from different social and health areas, as well as volunteers who provide a total of 24 services to the community on a daily basis. Since their establishment, the CdC model has proven to be more effective in attaining healthcare objectives in close proximity, such as achieving higher influenza vaccination coverage for target groups and facilitating diabetes follow-up [15].

The new conceptual framework for COPC implemented through the CdC model aims to strengthen the sense of affiliation between citizens and the CdC. CdCs function as nodes within the broader network of health, social, and social welfare services, while also serving as integral parts of local community living spaces. The objective is to place the community, including patients, caregivers, patient associations, and citizens, at the center, recognizing that healthcare is just one determinant of community well-being. The CdC strives to become an integral part of community identity, fostering participation and leveraging available resources. It empowers citizens and facilitates collaborative processes, such as co-programming and co-designing, to address various aspects, including accessibility, environmental characteristics, organizational issues, and continuity of care [1,8].

In this context, evaluating CdCs based on user experiences is a crucial aspect in understanding the impact of innovative care delivery models on the community’s perception of service quality. The study aims to analyze user perceptions of the CdC, highlighting any sociodemographic characteristics associated with these perceptions. As a result, this information can be used to customize healthcare services according to specific needs.

## 2. Materials and Methods

During the period of 13–18 March 2023, paper questionnaires were administered at the Rubicon Community Health Center (CdC). Upon entering the CdC, users were greeted by trained healthcare professionals (HCPs) and volunteers who were specifically trained to enroll participants. This occurred within the context of a meeting during which the study’s methods were thoroughly explained, and the intricate details of the survey were discussed comprehensively. The staff distributed the questionnaires, explained the purpose of the study, and provided assistance to users if needed. Posters were displayed in common areas to promote the data collection process. The completed questionnaires were collected when users exited the CdC. The inclusion criteria for the study were being 18 years of age or older and having the ability to understand written Italian. Participation in the study was voluntary, and the questionnaire was anonymous. The study was approved by the Bioethics Committee of the University of Bologna (Italy) on 3 March 2023 (protocol number 0058062).

To ensure that the sample was representative of the CdC user population, the working group identified specific time slots and days for conducting enrollment. This included both morning and afternoon hours, as well as weekdays and pre-holidays. The minimum sample size required was calculated to be 382 using a confidence level of 95%, margin of error 5%, population proportion 50% and population size 60,000. In order to achieve the desired level of representativeness and statistical power, a total of 865 questionnaires were distributed to reach the assumed sample size. The overall acceptance rate was 85.6%.

### 2.1. Questionnaire

The questionnaire was designed to assess the perceived quality of healthcare services at the CdC. It included both quantitative and qualitative sections. The quantitative section consisted of multiple-choice questions and four-point Likert scales, while the qualitative section comprised open-ended questions. The questionnaire aimed to collect information about users’ experiences with the provided services and was organized into various survey categories: accessibility and welcoming (availability of parking, public transportation, presence of a reception operator, and clear information), environment (facility maintenance conditions, cleanliness, presence of clear signs, facility’s welcoming atmosphere), staff (sensitivity, attention to values/cultures/traditions, ability to address specific health issues, clarity in providing health-related information), management (repeated need to describe one’s health condition to operators, receiving conflicting health opinions), and continuity of care (clear information about treatments, follow-up, retrieval of reports, health promotion, and activities conducted by volunteer associations at the facility).

The survey also inquired about the waiting experience and allowed users to provide an overall rating of their encounter with the services. The survey instrument was adapted from that used in a previous survey conducted by the Emilia-Romagna Region in 2018 at other COPC centers and Outpatient Clinics [14]. Some additional sociodemographic and facility-specific questions were also included, such as inquiries about the presence of voluntary associations within the CdC.

### 2.2. Analysis

The user ratings were analyzed by calculating frequency distributions for all satisfaction variables. Additionally, the rating scales of the questionnaires, ranging from 1 (not at all agree) to 4 (completely agree), were re-coded into two categories: “dissatisfied” (options 1 and 2) and “satisfied” (options 3 and 4).

To investigate the factors contributing to low overall satisfaction with the CdC’s services, a stepwise logistic regression analysis was conducted. This analysis aimed to determine the variables to be included in the final multiple logistic regression model, considering the principles of parsimony and biological plausibility. The age was included in the model as a single continuous variable. Regarding employment status, “being employed” was selected as the baseline class due to the substantial number of responses. For all other variables, the chosen baseline class was that which corresponded to the lowest level (of financial resources, educational level, distance from the CdC). The results of the multivariate analyses were presented as Odds Ratios (ORs) with corresponding 95% confidence intervals (95% CIs). Statistical significance was defined as *p* < 0.05.

Data collection was carried out using Microsoft Excel, while all analyses were performed using Stata 15 statistical software (StataCorp, College Station, TX, USA).

In this research, we utilized the qualitative methodology of content analysis to thoroughly explore the subtle details within the responses obtained from two open-ended questions in our questionnaire. This process involved a systematic collection and organization of data, fostering a comprehensive understanding. Using a specifically created code as a guide, each response was subjected to coding, resulting in a robust categorization framework.

## 3. Results

### 3.1. Sample Characteristics

Table 1 presents a complete overview of the sample, including valuable insights into the demographic characteristics, behaviors, and preferences of the respondents. A total of 741 individuals participated in the survey, with 37.9% identifying as female and an average age of 55.4 ± 16.2 years. Regarding cohabitation, the majority of participants (29.4%) reported living with two people, while 6.0% lived alone.

In terms of employment status, 52.7% were employed, 34.3% were retired, and 13.0% were unemployed. When asked about their financial resources, 37.8% mentioned that meeting their needs was easy, while 17.9% reported facing many difficulties. In terms of education, the largest proportion of respondents (37.0%) had a high school education, followed by middle school (33.4%) and university (14.3%).

The majority of respondents (78.3%) traveled to the CdC by car, and a smaller percentage arrived on foot (13.9%), by bike (4.8%) or by bus (1.1%). Approximately 70.5% of participants lived within a 15-min distance from the CdC. In terms of visit frequency, 57.9% reported visiting the CdC once or more per month, while 29.9% visited less than once per year. The primary reason for visiting the CdC was for personal healthcare needs (82.2%).

When asked about their awareness of the reporting process, 40.5% indicated that they knew how to make a report, while 59.5% did not. Furthermore, 66.9% of respondents had enabled the Electronic Health Record system. Finally, the services sought at the CdC varied, with the most common being the sampling point (20.6%) and the booking center (19.2%).

### 3.2. Healthcare Service Quality Perception

The survey results and insights into the respondents’ perceptions of various aspects related to accessibility, environment, staff, management, continuity of care, and overall satisfaction are shown in Table 2. In terms of accessibility and welcoming, the majority of respondents (43.5%) totally did not agree that parking availability was sufficient, while 41.6% strongly disagreed that the facility was easy to reach by public transportation.

However, a significant proportion (44.9%) totally agreed that there was a welcoming operator present, and 54.6% totally agreed that clear information was provided at the entrance. Regarding the environment, a large majority (45.8%) totally agreed that the facility was well-kept and well-maintained, and 52.1% totally agreed that it was clean. Additionally, 45.5% totally agreed that the facility had clear and understandable signs and 45% totally agreed that it was welcoming.

The staff was perceived positively, with a majority of respondents agreeing (32.3% to 51.4%) that they were sensitive, considered values/customs/traditions, considered individual needs and health problems, and provided adequate information about the respondent’s health condition.

The management of care received mixed responses, with varying opinions on care pathways, repetition of information, and differing opinions from professionals. In terms of continuity of care, a majority of respondents (56.7% to 63.2%) agreed or totally agreed that they had received complete information regarding treatment, follow-up checks, picking up reports, tips on staying in good health, and Volunteer Associations in the facility.

The overall ratings indicated that the majority of respondents (42.7% to 44.3%) agreed or totally agreed about the reliability and trustworthiness of the facility and found the overall quality to be satisfactory or totally satisfactory.

In terms of waiting, a significant proportion (46.5%) felt they had to wait a little for the service they used on the day of the questionnaire.

**Figure 1:**
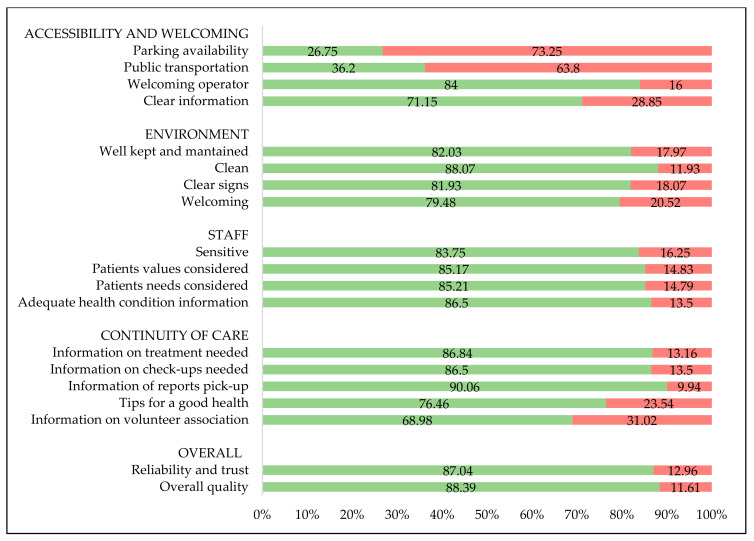
Levels of satisfaction (indicated in green) and dissatisfaction (indicated in red) among users of the CdC illustrated for survey categories: accessibility and welcoming, environment, staff, and continuity of care.

Figure 1 presents a graphical representation of the results shown in Table 2 regarding the perceived quality of CdC services divided into various categories.

### 3.3. Multivariate Analysis

The multivariate analysis findings from both Table 3 and Table 4 present odds ratios (OR) and their corresponding 95% confidence intervals (CI) for various variables related to the perceived quality questions included in the questionnaire. These variables include gender, age, employment status, financial resources, educational level, and distance from the CdC. The statistically significant associations between certain factors and the perceived quality of the CdC experience are highlighted in gray.

Table 3 displays the analysis of the “accessibility and welcoming”, “environment”, and “staff” categories.

The findings indicate that female participants were more likely to perceive the CdC as poorly maintained (OR: 1.94, 95% CI: 1.16–3.24), unclean (OR: 2.23, 95% CI: 1.19–4.17), and with insensitive staff (OR: 2.05, 95% CI: 1.21–3.46). Unemployed individuals tended to view the CdC as more welcoming (OR: 0.41, 95% CI: 0.19–0.87), while those with higher financial resources were more likely to perceive it as poorly connected to public transportation, poorly maintained, and unwelcoming when compared to respondents with very low financial resources.

Analyzing educational level, we found a significant statistical association for all questions related to the “environment” category, with higher ORs indicating a perception of poor quality among individuals with higher educational levels.

Furthermore, being more than 15 min away from the CdC was associated with a higher likelihood of perceiving lower quality in terms of public transportation (OR: 2.14, 95% CI: 1.16–3.96), insensitive professionals (OR: 1.63, 95% CI: 1.00–2.66), and inadequate health information provided by the healthcare staff (OR: 2.03, 95% CI: 1.12–3.54). Age did not consistently show significant associations with any of the “Accessibility and welcoming”, “environment”, and “staff” categories.

Table 4 presents the analysis of the “continuity of care” and “overall quality” categories.

Even when considering these two categories, the analysis of educational level revealed a significant statistical association. Individuals with higher educational levels were more likely to perceive lower reliability and trust in the CdC compared to those with lower educational levels (ranging from OR: 6.07, 95% CI: 1.34–27.51 for middle school to OR: 5.40, 95% CI: 1.03–28.40 for a university degree). Furthermore, being more than 15 min away from the CdC was associated with a higher likelihood of perceiving lower overall reliability and trust (OR: 1.74, 95% CI: 1.01–2.99).

The educational level also showed a significant statistical association regarding the enabling of the Electronic Health Record and knowledge of how to file a complaint report, with individuals with higher educational levels being more informed and aware (Table 4). In these case, older age was found to be associated with a lower likelihood of enabling the Electronic Health Record (OR: 0.97, 95% CI: 0.95–0.99), while older individuals were more likely to be aware of how to file a complaint report (OR: 1.03, 95% CI: 1.01–1.05).

### 3.4. Qualitative Analysis

At the conclusion of the questionnaire, participants were asked to provide suggestions for improving the quality of the CdC services. A total of 154 individuals (20.4% of the main sample) responded to this question, and their responses were subjected to qualitative analysis and grouped into thematic areas. The analysis revealed five main thematic categories:

- Parking: Some participants emphasized the need for additional parking spaces in the vicinity of the hospital. The difficulty in finding parking, which was also highlighted in the quantitative analysis, is particularly problematic on local market days.
- Waiting: Prolonged waiting times were identified as a concern for the booking center, specialist outpatient clinics, and general practitioners.
- Personnel: Respondents who utilized various services provided feedback on the behavioral and interpersonal aspects of healthcare professionals. Recommendations included the desire for more sincerity, active listening, empathy, understanding towards those who arrive late for appointments, kindness, clear communication, and sensitivity.
- Booking Center: This specific service received numerous suggestions, such as the need for additional counters, especially during lunch breaks, in order to expedite certain processes or address language barriers.
- Emergency Room: The need for an emergency room was highlighted in several responses, particularly due to its absence in previous years.

It is important to note that among the various open-ended responses, the CdC services also received multiple statements of praise.

## 4. Discussion

This study offers a comprehensive analysis of the demographic profiles, behaviors, and perceived healthcare quality among users of an Italian “Casa della Comunità” (Community-Oriented Primary Health Center). It delves into respondents’ perspectives on accessibility, environment, staff, management, continuity of care, and overall satisfaction. The results reveal a varied response from the participants across these dimensions.

In general, the majority of respondents conveyed their agreement regarding the reliability and trustworthiness of the CdC and expressed satisfaction with its overall quality. Positive perceptions of the environment indicated that the CdC has successfully established a welcoming and pleasant atmosphere for patients [16,17]. The presence of clear and comprehensible signs was also positively acknowledged, indicating effective communication within the facility. Additionally, respondents generally held positive perceptions of the staff across all domains, emphasizing the significance of patient-centered care and the crucial role healthcare professionals play in delivering satisfactory experiences to patients [18,19].

The management of care elicited diverse responses, reflecting differing opinions on care pathways, repetition of information, and conflicting viewpoints among professionals. These findings highlight potential areas for improvement, particularly in terms of care coordination and consistency in the information provided to patients. Enhancing communication and fostering collaboration among healthcare professionals can play a pivotal role in addressing these concerns and enhancing the overall management of care at the CdC [20]. Interprofessional collaboration in healthcare offers a multitude of benefits that extend beyond improved patient satisfaction. It also encompasses enhanced employee satisfaction and retention, reduced occurrence of medical errors and preventable complications, improved patient care and outcomes, decreased inefficiencies, lowered healthcare costs, and the ability to initiate treatment more promptly [20,21].

The study revealed several noteworthy associations, underscoring the impact of gender, financial resources, educational level, and proximity to the CdC (Community House) on the respondents’ perceptions. These findings suggest that sociodemographic factors significantly shape how individuals perceive the CdC’s services. By addressing these disparities and customizing services to cater to the unique needs and expectations of diverse demographic groups, it is possible to enhance the overall quality and accessibility of healthcare services [18,22].

The sample includes a diverse age range, which is essential for gaining insights into the experiences and perspectives of different generations. It is worth noting that there is a significant gender imbalance, with males outnumbering females. This disparity could potentially pose challenges in terms of accessing services and delivering appropriate care for female users, as confirmed by other scientific works [23,24]. This gender disparity may impede accessibility and effective support for female individuals, emphasizing the need to find solutions that bridge this gap [24].

The high unemployment rate and the significant number of participants experiencing financial difficulties in meeting their needs underscore the importance of recognizing economic disparities, as they have a profound impact on perceptions and expectations regarding healthcare services [22,25]. Specifically, in this study, unemployed individuals exhibited a tendency to perceive the CdC as more welcoming. Conversely, participants with higher financial resources were more prone to perceiving it as having limited connectivity to public transportation, inadequate maintenance, and an unwelcoming atmosphere.

The educational level of participants also displayed notable associations with perceptions of the environment, as higher educational levels were linked to a perception of poor quality. Previous studies have also highlighted the significance of respondents’ educational level as a crucial factor influencing health literacy, expectations, and their ability to navigate healthcare systems effectively [26,27].

Moreover, residing more than 15 min away from the CdC was found to be linked to a higher probability of perceiving lower quality in different aspects, including public transportation, insensitive professionals, and insufficient health information provided by the healthcare staff. Regarding accessibility, a significant number of respondents expressed dissatisfaction with the availability of parking and the convenience of reaching the facility via public transportation. The concept of proximity medicine, which aims to ensure that healthcare services are easily accessible to individuals, plays a crucial role in enhancing the quality of life for those with chronic health conditions. Existing literature indicates that proximity to healthcare services can improve access to high-quality care, resulting in fewer complications and improved overall outcomes [28]. The distance from the CdC is a particularly crucial factor to consider, especially in a context where the majority of users traveled by car, while smaller percentages arrived on foot or by bike. These findings emphasize the significance also of considering transportation connectivity to improve the overall accessibility of the CdC [29].

Finally, an intriguing finding from the study highlights a lack of awareness regarding the process of reporting potential gaps in communication and knowledge dissemination among the respondents. This lack of awareness could hinder the ability to provide effective feedback and address concerns. Considering this finding along with the substantial engagement in the survey, conducting ongoing monitoring studies on perceived quality holds significant importance. These studies contribute to obtaining a comprehensive understanding of the CdC and the needs of its users, thereby facilitating continuous enhancement.

In this specific case, the implications for the CdC involved sharing the results with key stakeholders engaged in the planning, organization, and delivery of healthcare services at the corporate level and within the facility. This dissemination aimed to enable organizational changes guided by the identified needs.

### Limitations

First and foremost, it should be noted that the questionnaire used in this study was exclusively available in the Italian language, posing a significant language barrier for individuals with limited proficiency in Italian. Although researchers and volunteers were present to provide assistance with questionnaire completion, this language limitation may have hindered participation. To address the technological barrier, a decision was made to administer a paper-based questionnaire instead of utilizing a digital format. However, this choice resulted in non-compulsory responses, allowing participants to skip certain questions. Furthermore, the length of the questionnaire may have contributed to non-responses, particularly towards the end of the survey. It is also important to acknowledge that, despite the questionnaires being collected anonymously, there is a possibility of introducing a desirability bias. Lastly, data collection was confined to a specific one-week period, and it is worth considering that the results obtained during this time may differ slightly from those obtained in other periods of the year. The length of the questionnaire, its availability only in the Italian language, and the administration concentrated within a single week could have potentially created a selection bias. This bias might have disadvantaged the participation of users with limited time availability, those who did not understand the Italian language, and those who utilized the services of the CdC during other specific periods of the year. As a result, this could have influenced the accuracy and applicability of the study’s findings.

Despite these limitations, efforts were made to diversify the collection times throughout different days and hours. The survey garnered substantial participation from CdC users, resulting in a sizable and diverse sample that aligns with verifiable local public data, such as unemployment rates.

## 5. Conclusions

In conclusion, our results provide valuable insights into the demographic characteristics, behaviors, and preferences of the CdC users, as well as their perceptions of the CdC health services quality. The findings highlight areas of strength, such as a welcoming environment, sensitive staff, and comprehensive continuity of care, while also pointing out areas for improvement, including parking availability, public transportation connectivity, waiting times, and consistency in care management. The associations between sociodemographic factors and perceived quality underscore the importance of considering individual characteristics and needs when designing and delivering healthcare services. These findings can inform strategies to enhance the quality, accessibility, and patient satisfaction at the CdC and similar healthcare facilities.

## Figures and Tables

**Table 1:** Sample characteristics.

Question, (n. of Respondents)		n (%)
Gender (n = 741)	Female	281 (37.9)
	Male	458 (61.8)
	Other	2 (0.3)
Age (n = 708)	55.4 ± 16.2	
Cohabitants (n = 728)	0	44 (6.0)
	1	141 (19.4)
	2	214 (29.4)
	3	155 (21.3)
	>3	174 (23.9)
Employment status (n = 731)	Employed	385 (52.7)
	Unemployed	95 (13.0)
	Retired	251 (34.3)
With your financial resources can you meet your needs? (n = 703)	Very easily	80 (11.4)
	Easily	266 (37.8)
	With some difficulties	231 (32.9)
	With many difficulties	126 (17.9)
Educational Level (n = 743)	Primary school or less	96 (12.9)
	Middle school	248 (33.4)
	High school	275 (37.0)
	University	106 (14.3)
	Post-graduate	18 (2.4)
By what means did you come? (n = 742)	Car	581 (78.3)
	On foot	103 (13.9)
	Bike	36 (4.8)
	Motorcycle	6 (0.8)
	Bus	8 (1.1)
	Other	8 (1.1)
How far is the CdC from where you live? (n = 709)	<15 min	500 (70.5)
	>15 min	209 (29.5)
How often do you visit the CdC? (n = 693)	Once a week	41 (5.9)
	More than once a week	44 (6.3)
	Once or more per month	401 (57.9)
	Less than once per year	207 (29.9)
Who did you come to the CdC for? (n = 698)	For myself	574 (82.2)
	For other people	124 (17.8)
In case you wanted to make a report (complaint, commendation, suggestion), would you know how to do it? (n = 688)	No	409 (59.5)
	Yes	279 (40.5)
Have you enabled the Electronic Health Record? (n = 704)	No	233 (33.1)
	Yes	471 (66.9)
What service did you come for today? (n = 683)	Sampling point *	141 (20.6)
	Booking center **	131 (19.2)
	Specialist outpatient clinic	83 (12.2)
	General pratictioner	82 (12.0)
	Radiology service	58 (8.5)
	Other	188 (27.5)

* Sampling point: sample collection for laboratory sampling; ** Booking center: healthcare appointment facility.

**Table 2:** Healthcare service quality perception.

Survey Category	Question, (n. of Respondents)		n (%)
**Accessibility and welcoming**	Parking availability (n = 643)	Strongly disagree	280 (43.5)
		Disagree	191 (29.7)
		Agree	118 (18.4)
		Strongly agree	54 (8.4)
	Easy to reach by public transportation (n = 279)	Strongly disagree	116 (41.6)
		Disagree	62 (22.2)
		Agree	49 (17.6)
		Strongly agree	52 (18.6)
	Presence of an operator who welcomes (n = 558)	Strongly disagree	74 (13.3)
		Disagree	87 (15.6)
		Agree	146 (26.2)
		Strongly agree	251 (44.9)
	Clear information at the entrance (n = 626)	Strongly disagree	43 (6.9)
		Disagree	72 (11.5)
		Agree	169 (27)
		Strongly agree	342 (54.6)
**Environment**	Well-kept and well-maintained (n = 651)	Strongly disagree	28 (4.3)
		Disagree	89 (13.7)
		Agree	236 (36.2)
		Strongly agree	298 (45.8)
	Clean (n = 654)	Strongly disagree	24 (3.7)
		Disagree	54 (8.3)
		Agree	235 (35.9)
		Strongly agree	341 (52.1)
	With clear and understandable signs (n = 631)	Strongly disagree	31 (4.9)
		Disagree	83 (13.1)
		Agree	230 (36.5)
		Strongly agree	287 (45.5)
	Welcoming (n = 653)	Strongly disagree	29 (4.4)
		Disagree	105 (16.1)
		Agree	225 (34.5)
		Strongly agree	294 (45)
**Staff**	Sensitive (n = 646)	Strongly disagree	27 (4.2)
		Disagree	78 (12.1)
		Agree	209 (32.3)
		Strongly agree	332 (51.4)
	Considers your values, customs, and traditions (n = 518)	Strongly disagree	27 (4.7)
		Disagree	58 (10.1)
		Agree	127 (31.8)
		Strongly agree	306 (53.4)
	Considers your needs and the specificity of your health problems (n = 514)	Strongly disagree	24 (4.7)
		Disagree	52 (10.1)
		Agree	168 (32.7)
		Strongly agree	270 (52.5)
	Adequate information about your health condition (n = 600)	Strongly disagree	30 (5)
		Disagree	51 (8.5)
		Agree	191 (31.8)
		Strongly agree	328 (54.7)
**Management**	Care pathway user (n = 45)	Palliative care	12 (26.7)
		Heart Failure Nursing Outpatient Clinic	5 (11.1)
		Diabetes Nursing Outpatient Clinic	28 (62.2)
	I did not have to repeat the same things and health information to everyone all the time (n = 44)	Strongly disagree	11 (25)
		Disagree	4 (9.1)
		Agree	9 (20.5)
		Strongly agree	20 (45.4)
	I have received differing opinions from different professionals on the same topics (n = 41)	Strongly disagree	15 (36.6)
		Disagree	7 (17.1)
		Agree	5 (12.2)
		Strongly agree	14 (34.1)
**Continuity of care**	I received complete information regarding treatment needed (n = 494)	Strongly disagree	20 (4)
		Disagree	45 (9.1)
		Agree	149 (30.2)
		Strongly agree	280 (56.7)
	I received complete information regarding follow-up checks needed (n = 489)	Strongly disagree	25 (5.1)
		Disagree	41 (8.4)
		Agree	143 (29.2)
		Strongly agree	280 (57.3)
	I received complete information regarding how to pick up the reports (n = 513)	Strongly disagree	19 (3.7)
		Disagree	32 (6.2)
		Agree	138 (26.9)
		Strongly agree	324 (63.2)
	I received complete information regarding tips on how to stay ingood health (n = 429)	Strongly disagree	36 (8.4)
		Disagree	65 (15.2)
		Agree	138 (32.1)
		Strongly agree	190 (44.3)
	I received complete information regarding Volunteer Associations in the CdC (n = 374)	Strongly disagree	60 (16)
		Disagree	56 (15)
		Agree	113 (30.2)
		Strongly agree	145 (38.8)
**Overall rating**	Reliability and trust (n = 648)	Strongly disagree	14 (2.2)
		Disagree	70 (10.8)
		Agree	277 (42.7)
		Strongly agree	287 (44.3)
	Overall quality (n = 663)	Not at all satisfactory	16 (2.4)
		Unsatisfactory	61 (9.2)
		Satisfactory	314 (47.4)
		Totally satisfactory	272 (41)
**Waiting**	For the service used on the day the questionnaire was filled out (n = 681)	Not at all	102 (15)
		A little	317 (46.5)
		Significant	206 (30.3)
		A lot	56 (8.2)

**Table 3:** Multivariate analysis identifying factors associated with lower perceived quality within each survey category: “accessibility and welcoming”, “environmental”, and “staff”. Baseline classes are denoted by a value of 1.

	Accessibility and Welcoming				Environment				Staff			
	Parking Availability	Public Transportation	Welcoming Operator	Clear Information	Well Kept and Mantained	Clean	Clear Signs	Welcoming	Sensitive	Patients Values Considered	**Patients Needs Considered**	**Adequate Health Information**
	OR (95% CI)	OR (95% CI)	OR (95% CI)	OR (95% CI)	OR (95% CI)	OR (95% CI)	OR (95% CI)	OR (95% CI)	OR (95% CI)	OR (95% CI)	**OR (95% CI)**	**OR (95% CI)**
**Gender**												
Female	1.26 (0.85–1.87)	1.02 (0.57–1.83)	0.70 (0.76–1.78)	0.93 (0.58–1.47)	1.94 (1.16–3.24)	2.23 (1.19–4.17)	1.02 (0.64–1.63)	1.13 (0.72–1.76)	2.05 (1.21–3.46)	1.40 (0.80–2.47)	1.01 (0.57–1.76)	1.24 (0.70–2.18)
**Age**	0.99 (0.97–1.01)	1.02 (1.00–1.04)	0.99 (0.97–1.01)	0.99 (0.97–1.01)	1.00 (0.97–1.02)	1.00 (0.97–1.02)	1.00 (0.97–1.01)	1.00 (0.97–1.01)	0.98 (0.96–1.00)	0.99 (0.96–1.01)	0.99 (0.96–1.01)	0.98 (0.95–1.00)
Employment status												
Employed	1	1	1	1	1	1	1	1	1	1	1	1
Unemployed	0.97 (0.54–1.77)	0.60 (0.26–1.37)	0.59 (0.30–1.15)	0.57 (0.26–1.24)	0.76 (0.37–1.58)	0.60 (0.24–1.50)	0.60 (0.28–1.31)	0.41 (0.19–0.87)	0.87 (0.43–1.77)	0.74 (0.34–1.61)	0.70 (0.30–1.64)	0.57 (0.24–1.37)
Retired	1.46 (0.78–2.74)	0.97 (0.40–2.38)	1.71 (0.87–3.36)	1.54 (0.74–3.20)	1.61 (0.77–3.39)	1.78 (0.74–4.24)	1.26 (0.60–2.66)	0.93 (0.45–1.90)	1.36 (0.64–2.91)	1.15 (0.48–2.73)	0.97 (0.39–2.41)	1.77 (0.74–4.23)
**Financial resources**												
Very low	1	1	1	1	1	1	1	1	1	1	1	1
Low	1.33 (0.71–2.50)	3.52 (1.32–9.36)	2.15 (0.98–4.71)	1.83 (0.83–4.03)	3.99 (1.35–11.75)	2.37 (0.78–7.23)	1.78 (0.78–4.05)	2.91 (1.17–7.22)	0.94 (0.45–1.96)	1.48 (0.57–3.81)	1.26 (0.48–3.32)	3.32 (0.96–11.56)
High	1.36 (0.71–2.60)	3.09 (1.14–8.35)	2.05 (0.91–4.65)	1.01 (0.43–2.36)	2.64 (0.87–8.00)	2.03 (0.65–6.32)	1.28 (0.54–3.03)	2.05 (0.80–5.23)	0.70 (0.32–1.50)	1.10 (0.41–2.95)	1.02 (0.38–2.78)	2.00 (0.57–7.22)
Very high	1.27 (0.61–2.68)	2.20 (0.73–6.61)	2.86 (1.16–7.07)	1.93 (0.76–4.87)	3.08 (0.94–10.06)	1.43 (0.40–5.20)	1.09 (0.40–2.96)	2.52 (0.92–6.95)	0.53 (0.21–1.31)	1.23 (0.42–3.62)	1.58 (0.54–4.66)	3.59 (0.96–13.43)
**Educational level**												
Primary school or less	1	1	1	1	1	1	1	1	1	1	1	1
Middle school	1.22 (0.61–2.46)	0.75 (0.24–2.36)	1.33 (0.60–2.93)	2.44 (0.92–6.47)	4.42 (1.43–13.73)	3.42 (0.92–12.69)	3.18 (1.03–9.88)	2.55 (0.90–7.25)	1.23 (0.51–2.97)	2.14 (0.66–6.93)	1.33 (0.47–3.75)	1.77 (0.54–5.82)
High school	1.29 (0.62–2.71)	0.90 (0.28–2.89)	1.68 (0.75–3.77)	2.67 (0.97–7.32)	3.92 (1.22–12.60)	4.32 (1.12–16.34)	3.47 (1.09–11.06)	3.32 (1.14–9.70)	1.07 (0.43––2.67)	2.40 (0.72–8.03)	1.24 (0.41–3.76)	2.96 (0.89–9.87)
University	1.10 (0.47–2.59)	0.82 (0.22–3.04)	1.43 (0.56–3.66)	2.34 (0.75–7.30)	5.85 (1.66–20.55)	4.92 (1.14–21.18)	3.77 (1.07–13.22)	3.51 (1.10–11.21)	0.98 (0.34–2.79)	1.41 (0.36–5.51)	0.52 (0.13–2.04)	1.53 (0.38–6.16)
**Distance**												
<15 min	1	1	1	1	1	1	1	1	1	1	1	1
>15 min	0.82 (0.54–1.23)	2.14 (1.16–3.96)	0.94 (0.60–1.47)	0.89 (0.54–1.47)	1.48 (0.90–2.43)	1.44 (0.82–2.56)	1.38 (0.84–2.25)	1.37 (0.86–2.16)	1.63 (1.00–2.66)	1.42 (0.81–2.47)	1.14 (0.63–2.05)	2.03 (1.12–3.54)

A gray background color was used to highlight values that demonstrate statistical significance.

**Table 4:** Multivariate analysis identifying factors associated with lower perceived quality within each survey category: “continuity of care”, “overall satisfaction”) and predictors of “Enabling of the Electronic Health Record” and “Knowledge of how to file a complaint report”. Baseline classes are denoted by a value of 1.

	Continuity of Care					Overall			
	Information on Treatment Needed	Information on Check-Ups Needed	Information of Reports Pick-Up	Tips for a Good Health	Information on Volunteer Association	Reliability and Trust	Overall Quality	Electronic Health Record Enabled	**Knowledge of How to File a Complaint Report**
	OR (95% CI)	OR (95% CI)	OR (95% CI)	OR (95% CI)	OR (95% CI)	OR (95% CI)	OR (95% CI)	OR (95% CI)	**OR (95% CI)**
**Gender**									
Female	1.15 (0.63–2.11)	1.38 (0.76–2.50)	1.28 (0.65–2.51)	1.03 (0.62–1.71)	1.29 (0.77–2.18)	0.92 (0.54–1.55)	0.89 (0.51–1.56)	0.77 (0.51–1.16)	0.91 (0.64–1.30)
**Age**	1.00 (0.97–1.03)	1.00 (0.97–1.03)	1.01 (0.98–1.04)	0.98 (0.96–1.01)	0.98 (0.96–1.01)	0.99 (0.97–1.02)	1.00 (0.97–1.02)	0.97 (0.95–0.99)	1.03 (1.01–1.05)
Employment status									
Employed	1	1	1	1	1	1	1	1	1
Unemployed	0.65 (0.26–1.63)	0.51 (0.21–1.24)	0.53 (0.19–1.45)	1.04 (0.50–2.16)	0.57 (0.26–1.21)	0.39 (0.15–1.06)	0.45 (0.17–1.21)	0.74 (0.39–1.40)	0.81 (0.47–1.39)
Retired	1.08 (0.40–2.88)	0.61 (0.23–1.63)	0.42 (0.14–1.31)	1.47 (0.63–3.44)	1.01 (0.42–2.45)	1.36 (0.60–3.11)	0.87 (0.35–2.19)	0.72 (0.38–1.35)	0.78 (0.44–1.37)
**Financial resources**									
Very low	1	1	1	1	1	1	1	1	1
Low	1.73 (0.55–5.44)	1.05 (0.36–3.04)	1.88 (0.52–6.78)	1.55 (0.58–4.15)	0.91 (0.37–2.24)	1.68 (0.66–4.31)	2.61 (0.76–9.04)	1.17 (0.60–2.31)	1.21 (0.68–2.17)
High	0.96 (0.29–3.23)	0.72 (0.24–2.17)	0.97 (0.25–3.75)	1.22 (0.44–3.42)	0.84 (0.33–2.13)	1.35 (0.51–3.58)	3.00 (0.86–10.48)	0.73 (0.37–1.46)	1.11 (0.61–2.02)
Very high	2.73 (0.79–9.43)	1.94 (0.62–6.08)	1.57 (0.37–6.61)	2.16 (0.72–6.42)	1.45 (0.51–4–15)	1.71 (0.59–4.95)	3.09 (0.80–11.92)	0.81 (0.38–1.75)	1.02 (0.52–2.01)
**Educational level**									
Primary school or less	1	1	1	1	1	1	1	1	1
Middle school	1.00 (0.33–3.01)	0.98 (0.33–2.94)	1.42 (0.35–5.79)	1.96 (0.67–5.72)	0.77 (0.30–1.96)	6.07 (1.34–27.51)	1.71 (0.52–5.70)	1.88 (0.97–3.65)	2.35 (1.24–4.48)
High school	1.26 (0.41–3.91)	1.04 (0.33–3.29)	1.22 (0.29–5.24)	2.31 (0.75–7.12)	1.49 (0.56–3.91)	5.83 (1.24–27.36)	1.48 (0.42–5.22)	4.31 (2.12–8.75)	2.01 (1.02–3.96)
University	1.15 (0.31–4.20)	0.71 (0.19–2.67)	0.63 (0.12–3.44)	1.63 (0.46–5.73)	1.07 (0.35–3.25)	5.40 (1.03–28.40)	1.62 (0.40–6.53)	4.76 (1.93–11.72)	2.66 (1.21–5.85)
Post-graduate	3.39 (0.56–20.59)	1.17 (0.16–8.31)	2.15 (0.25–18.70)	3.38 (0.61–18.62)	1.12 (0.23–5.62)	7.57 (0.84–68.34)	2.00 (0.27–14.57)	1	3.71 (0.98–13.97)
**Distance**									
<15 min	1	1	1	1	1	1	1	1	1
>15 min	1.42 (0.77–2.62)	1.31 (0.72–2.39)	1.09 (0.54–2.19)	0.90 (0.51–1.57)	0.72 (0.41–1.27)	1.74 (1.01–2.99)	1.17 (0.65–2.12)	1.22 (0.78–1.90)	0.90 (0.62–1.31)

A gray background color was used to highlight values that demonstrate statistical significance.

## Data Availability

The data presented in this study are available on request from the corresponding author.
